# Supplementary material for: Overcoming Challenges in Pediatric Formulation with a Patient-Centric Design Approach: A Proof-of-Concept Study on the Design of an Oral Solution of a Bitter Drug
Source: Pharmaceuticals (Basel). 2022 Oct 27;15(11):1331. doi: 10.3390/ph15111331 (PMC9694284; doi:10.3390/ph15111331)
Supplement: Supplementary file 1 [file pharmaceuticals-15-01331-s001.zip › pharmaceuticals-1966126-supplementary.pdf]

Supplementary Material

Figure S1 Composition of the 20 lipid polymeric cross-sensitive sensor membranes (40 sensors in total),

- S1:1 or S2:1 - 3% of of octdecylamine + 32% of bis(1-butylpentyl) adipate + 65% of polyvinyl chloride;
- S1:2 or S2:2 - 3% of of oleyl alcohol + 32% of bis(1-butylpentyl) adipate + 65% of polyvinyl chloride;
- S1:3 or S2:3 - 3% of of methyltrioctylammonium chloride + 32% of bis(1-butylpentyl) adipate + 65% of polyvinyl chloride;
- S1:4 or S2:4 - 3% of of oleic acid + 32% of bis(1-butylpentyl) adipate + 65% of polyvinyl chloride;
- S1:5 or S2:5 - 3% of of octdecylamine + 32% of dibutyl sebacate + 65% of polyvinyl chloride;
- S1:6 or S2:6 - 3% of of oleyl alcohol + 32% of dibutyl sebacate + 65% of polyvinyl chloride;
- S1:7 or S2:7 - 3% of methyltrioctylammonium chloride + 32% of dibutyl sebacate + 65% of polyvinyl chloride;
- S1:8 or S2:8 - 3% of oleic acid + 32% of dibutyl sebacate + 65% of polyvinyl chloride;
- S1:9 or S2:9 - 3% of octdecylamine + 32% of 2-nitrophenyl-octyl ether + 65% of polyvinyl chloride;
- S1:10 or S2:10 - 3% of oleyl alcohol + 32% of 2-nitrophenyl-octyl ether + 65% of polyvinyl chloride;
- S1:11 or S2:11 - 3% of methyltrioctylammonium chloride + 32% of 2-nitrophenyl-octyl ether + 65% of polyvinyl chloride;
- S1:12 or S2:12 - 3% of oleic acid + 32% of 2-nitrophenyl-octyl ether + 65% of polyvinyl chloride;
- S1:13 or S2:13 - 3% of octdecylamine + 32% of tris(2-ethylhexyl) phosphate + 65% of polyvinyl chloride;
- S1:14 or S2:14 - 3% of oleyl alcohol + 32% of tris(2-ethylhexyl) phosphate + 65% of polyvinyl chloride;
- S1:15 or S2:15 - 3% of methyltrioctylammonium chloride + 32% of tris(2-ethylhexyl) phosphate + 65% of polyvinyl chloride;
- S1:16 or S2:16 - 3% of oleic acid + 32% of tris(2-ethylhexyl) phosphate + 65% of polyvinyl chloride;
- S1:17 or S2:17 - 3% of octdecylamine + 32% of dioctyl phenylphosphonate + 65% of polyvinyl chloride;
- S1:18 or S2:18 - 3% of oleyl alcohol + 32% of dioctyl phenylphosphonate + 65% of polyvinyl chloride;
- S1:19 or S2:19 - 3% of methyltrioctylammonium chloride + 32% of dioctyl phenylphosphonate + 65% of polyvinyl chloride;
- S1:20 or S2:20 - 3% of oleic acid + 32% of dioctyl phenylphosphonate + 65% of polyvinyl chloride.

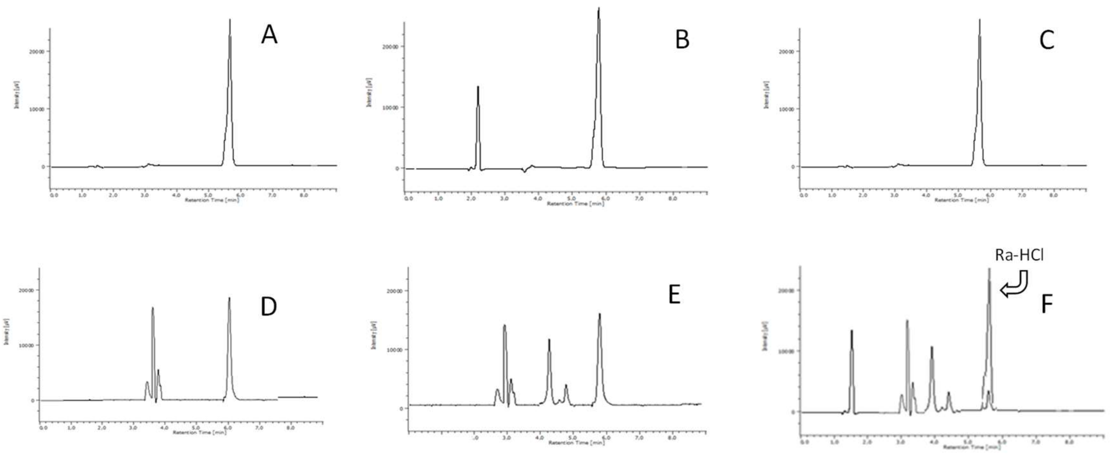

Figure S2 Chromatograms of degradation products at in the A) acid, B) base, C) temperature, D) UVA, E) peroxide, F) overlap all chromatograms.
